# AHLs Regulate Biofilm Formation and Swimming Motility of *Hafnia alvei* H4

**DOI:** 10.3389/fmicb.2019.01330

**Published:** 2019-06-19

**Authors:** Yao lei Zhu, Hong man Hou, Gong liang Zhang, Yi fang Wang, Hong shun Hao

**Affiliations:** ^1^School of Food Science and Technology, Dalian Polytechnic University, Dalian, China; ^2^Liaoning Key Lab for Aquatic Processing Quality and Safety, Dalian, China

**Keywords:** *Hafnia alvei*, AHLs, quorum sensing, biofilm, swimming motility

## Abstract

The aim of this study was to evaluate the role of *N*-acyl homoserine lactones (AHLs) in the regulation of swimming motility of *Hafnia alvei* H4 and its biofilm formation on 96-well plate, glass and stainless-steel surfaces. The *luxI* gene, which codes for an enzyme involved in AHL synthesis, was deleted to generate a *luxI* mutant (Δ*luxI*). The mutant produced no AHL, and the relative expression of the *luxR* gene was significantly (*P* < 0.05) decreased. In addition, *q*RT-PCR analysis showed that the relative expression of the *luxR* gene in Δ*luxI* was stimulated by the presence of exogenous AHLs (C4-HSL, C6-HSL, and 3-o-C8-HSL) added at concentrations ranging from of 50–250 μg/ml. Among the three AHLs, C6-HSL had the strongest effect. The ability of Δ*luxI* to form biofilm on 96-well plate, glass and stainless-steel surfaces was significantly reduced (*P* < 0.05) compared with the wild type (WT), but was increased when provided with 150 μg/ml C4-HSL, whereas C6-HSL and 3-o-C8-HSL had no effect. Scanning electron microscopy analysis of the biofilm revealed less bacteria adhering to the surface of stainless-steel and fewer filaments were found binding to the cells compared with the WT. Furthermore, Δ*luxI* also exhibited significant (*P* < 0.05) decrease in the expression of biofilm- and swimming motility-related genes, *flgA*, *motA* and *cheA*, consistent with the results observed for biofilm formation and swimming motility. Taken together, the results suggested that in *H. alvei* H4, C4-HSL may act as an important molecular signal through regulating the ability of the cells to form biofilm, as well as through regulating the swimming motility of the cell, and this could provide a new way to control these phenotypes of *H. alvei* in food processing.

## Introduction

Quorum sensing (QS) is a cell-to-cell communication system used by bacteria, and it is widely by both Gram-negative and Gram-positive bacteria (Ammor et al., 2008). Bacteria secret several kinds of chemical compounds that can act as signaling molecules [autoinducers (AIs)]. *N*-acyl homoserine lactone (AHL), also known as AI-1, is secreted by Gram-negative bacteria, and the communication mechanism of this compound involves AHL synthase (LuxI) and the transcription factor LuxR, which is responsible for controlling gene expression in the presence of AHLs. The LuxI/LuxR system has become the model system of AHLs-mediated quorum sensing, and the quorum-sensing system of Gram-negative is based on this system. LuxI synthesizes AHLs and it is encoded by the *luxI* gene. LuxR is encoded by the *luxR* gene, and it acts by binding to AHLs, thereby stimulating the expression of these genes in the presence of AHLs. The LuxI/LuxR complex is responsible for the up- or down-regulation of multiple target genes, such as those that code for pectinase, cellulase, and protease (Swift et al., 2001). Autoinducer-2 (AI-2) is synthesized from 4,5-dihydroxy-2,3-pentanedione (DPD) by LuxS, and it is used by Gram-negative and Gram-positive bacteria in interspecies communication. Peptides and derived peptides, generally serve as signaling molecules in Gram-positive bacteria (Bai and Rai, 2011).

Biofilm is a bacterial self-protection growth pattern and it is formed by the aggregation of bacterial cells within an extracellular matrix, which is mainly made of exopolimers (EPS) (Wang J. et al., 2016), and the adherence of bacterial cells to a solid surface depends on the EPS that the cells secret (Jung et al., 2013). In general, some pathogens and spoilage bacteria can adhere to the solid surfaces that can come into contact with food, such as the surfaces of food processing machines and packaging materials. These bacteria may then form biofilms, and the biofilms will allow the cells to become more resistant to cleaning treatments, and enable them to contaminate the food during subsequent processing (Gounadaki et al., 2008; Bai and Rai, 2011). This will effectively facilitate the transmission of the bacteria to the consumers via the contaminated food, eventually causing infections. Biofilms have been recognized as a frequent source of bacterial infections (Costerton et al., 1999). According to a report by Janssens et al. (2008), nearly 80% of persistent bacterial infections in the US were found to be related to biofilms. The formation of biofilm is a multi-step process, which consists of initial attachment, irreversible attachment, early development of biofilm architecture (microcolony formation), maturation and dispersion (Srey et al., 2013). Quorum sensing appears to be involved in all the steps of the process. Promotion and inhibition of biofilm formation by exogenous AHLs have been reported for *Shewanella baltica* (Zhao et al., 2016), *Serratia* A2 and *Aeromonas* B1 (Zhang et al., 2016), *Vibrio parahaemolyticus* (Bai and Rai, 2016), and *Pseudomonas* sp. HF-1 (Wang et al., 2012), suggesting that QS has a regulatory role in biofilm formation.

*H. alvei* is a Gram-negative, short-rod-shaped, flagellated bacterium that belongs to the family *Enterobacteriaceae*, which is considered as an opportunistic pathogen of humans and animals (Tan et al., 2014). However, despite being classified as a member of the *Enterobacteriaceae* family, *H. alvei* is still far from being virulent and pathogenic (Vivas et al., 2008). *H. alvei* is a common bacterial food contaminant (Liu et al., 2006), and it has been frequently isolated from spoiled food products, especially in chill-stored proteinaceous raw food, like refrigerated spherical fish paste (Tan et al., 2014), vacuum-packed beef (Bruhn et al., 2004) and raw milk (Viana et al., 2009). The strong tendency of *H. alvei* to adhere to solid surface and to form biofilm has been reported by Viana et al. (2009) and Hou et al. (2017), and it is considered to be a potentially important factor that causes food contamination and food spoilage. Therefore, it is necessary to look for effective ways to control biofilm formation. To our knowledge, fewer studies have studied the regulatory mechanism of quorum sensing of *H. alvei* with respect to biofilm formation and the motility of the cells in an artificial medium. Understanding more about the mechanism by which quorum sensing can impact biofilm formation will open up a new way to tackle the problem of food contamination by bacteria, and help safeguard better food quality and prevent food-poising.

In our previous study, we isolated a strain of *H. alvei* (*H. alvei* H4) from spoiled instant sea cucumber, and identified three kinds of AHLs secreted by this bacterium. These AHLs are C4-HSL, C6-HSL, and 3-o-C8-HSL. In addition, we also detected a significant influence of AHLs on the biofilm formation of *H. alvei* H4 (Hou et al., 2017). In this study, a *luxI* mutant of *H. alvei* H4 was constructed to conduct further research on the regulatory roles of C4-HSL, C6-HSL, and 3-o-C8-HSL in biofilm formation and swimming motility of *H. alvei* H4.

## Materials and Methods

### Bacterial Strains and Culture Conditions

The bacterial strains used in this study are presented in **Table 1**. *Chromobacterium violaceum* CV026, and *H. alvei* H4 were routinely cultured at 30°C, while *Escherichia coli* was grown at 37°C. All strains were grown in LB medium (Luria Bertani, 10 g/l tryptone, 5 g/l yeast extract, 10 g/l NaCl) supplemented with antibiotics where appropriate (50 μg/ml ampicillin and 34 μg/ml chloramphenicol in the case of *E. coli* culture or 20 μg/ml chloramphenicol for the *H. alvei* H4 mutant).

**Table 1:** Strains and plasmids used in this study.

Strains and plasmids	Relevant characteristic(s)	Source/references
*C. violaceum* CV026	mini-Tn-5 mutant of ATCC31532, violacein reporter, Km^R^	McClean et al. (1997)
*H. alvei* H4	Wild type	This study
Δ*luxI*	H4 derivative, *ΔluxI*::Cm^R^	This study
*E. coli*β2155	Cm^R^	Purchased from Takara
*E. coli* TOP10	General cloning strain, Str^R^	Purchased from Takara
PCVD442	Suicide plasmid, SacB, oriT, Am^R^	Songon
Puc19	Am^R^	Purchased from Takara

### Construction of Δ*luxI* Strain

To construct a *luxI-*deficient strain of *H. alvei* H4, a chloramphenicol resistance marker (Cm^R^) was inserted into the genomic DNA of *H. alvei* H4 at the *luxI* locus. Briefly, a 608-bp upstream homologous recombination arm and a 654-bp downstream homologous recombination arm of the *luxI* gene were amplified from the gDNA of *H. alvei* H4 and then cloned into the plasmid pUC19 to yield the construct pUC19-Δ*luxI.* The two DNA fragments were linked by a *Bam*HI restriction site. The chloramphenicol resistance maker (Cm^R^) was amplified from the plasmid pKD3, and then cloned into pUC19-Δ*luxI* at a site between the upstream and downstream homologous recombination arm fragments, yielding the construct pUC19-Δ*luxI*::Cm. Target fragment in pUC19-Δ*luxI*::Cm was subcloned and ligated into the suicide plasmid pCVD442 to yield the construct pCVD442-Δ*luxI*::Cm, which was then introduced into *E. coli*β2155 by electroplating. Conjugation between *E. coli*β2155 harboring pCVD442-Δ*luxI*::Cm and *H. alvei* H4 was then performed. The mutant colonies obtained were verified by PCR and DNA sequencing. Primers used in this section see **Table 2**.

**Table 2:** Primers used for the construction of Δ*luxI.*

Primer name	Primer sequences
Upstream of *luxI*	ATAGAATTCGTCGACATCACATTGATGTCAGACCTCAAGATTTC (*Eco*RI-*Sal*I)
	ATAGGATCCATATCTGAGTGAGGATGAGCGAATTTATC (*Bam*HI)
Downstream of *luxI*	ATAGGATCCATCACCTTGATTACATTTTAGTTACTATCC (*Bam*HI)
	ATAGCATGCGTCGACTACTGCCCTTGCTTTCTCAATGAC (*Sph*I-*Sal*I)
Chloramphenicol resistance gene region	ATAGGATCCATATGAATATCCTCCTTAGTTCCTATTC (*Bam*HI)
	ATAGGATCCGAGCTGCTTCGAAGTTCCTA (*Bam*HI)

*The underlined are restriction sites*.

### AHLs Production

*Chromobacterium violaceum* CV026 was used as the biosensor strain to detect the production of AHLs by *H*. *alvei* H4 WT and Δ*luxI*, since in the presence of AHLs, *C. violaceum* CV026 would produce a purple pigment that could be easily detected. This assay was performed as described by Viana et al. (2009). Briefly, AHLs were extracted from the corresponding bacterial culture. Aliquot (100 ml) of an overnight culture was centrifuged at 8000 ×*g* for 15 min at 4°C, and the supernatant was added to an equal volume of ethyl acetate containing 0.1% acetic acid (v/v) followed by thorough mixing. The mixture was then incubated at 25°C for 2 h with shaking at 180 rpm. After that, the ethyl acetate layer was removed and freeze-dried under vacuum, and the residue was dissolved in 1 ml ultra-pure water. To prepare the plates for the assay, 80 ml LB agar medium was cooled to about 60°C, mixed with 20 ml overnight culture of CV026, and then poured into sterile plates (20 ml per plate). Holes were punched into the solidified medium at the center of each plate using a sterile 1 ml pipette tip, and 60 μl of the AHL extract was dispensed into the hole. The plates was incubated at 30°C until the purple zone appeared around the point of AHL application.

### Biofilm Formation on 96-Well-Plate

Biofilm formation was measured using the microplate assay as described by Hou et al. (2017), but with some modifications. Briefly, culture of *H. alvei* H4 WT and Δ*luxI* were grown to an OD_600__nm_ value of 1.0, and then diluted 100-fold in LB medium. The diluted culture was dispensed into a 96-well plate polypropylene microtiter plate (Corning, NY, United States) using 200 μl per well. After incubation at 30°C for 24 h, the OD_600__nm_ of the culture was measured. After that, the culture medium was removed and washed off unattached cells by washing each well with 250 μl of 10 mM PBS (pH 7.2). A total of three washes were performed. This was followed by the addition of 250 μl anhydrous methanol and a 15-min incubation to fix the cells. Subsequently, 250 μl of 0.1% (v/v) crystal violet solution was added to each well and the plate was incubated at room temperature for 15 min and rinsed three times with deionized water (250 μl per rinse). The crystal violet was dissolved by the addition of 200 μl 33% glacial acetic acid followed by shaking at 300 rpm for 15 min. Biofilm formation was finally analyzed by measuring the absorbance of the plate at OD_590__nm_ using a Spectra M2 spectrophotometer (Molecular Devices, United States). Biofilm formed by Δ*luxI* in the presence of 150 μg/ml C4-HSL, 200 μg/ml C6-HSL, or 100 μg/ml 3-o-C8-HSL was detected essentially as described above.

### Biofilm Formation on Glass Surface

To examine the effect of quorum sensing on biofilms formed by *H. alvei* H4 on the glass surface, biofilm formation was investigated as described by Cai et al. (2018) with some modifications. Briefly, an overnight bacterial culture was diluted 100-fold in fresh LB medium, and 2 ml of the diluted culture was added to a glass tube (1 cm × 10 cm). As a control, 2 ml of LB was added to a separate glass tube. Both tubes were incubated at 30°C for 24 h without shaking. After incubation, the culture medium was gently removed, and each tube was washed three times with 2.5 ml PBS, and then with 2.5 ml anhydrous methanol followed by drying at 60°C for 15 min. The tube was then stained with 2.5 ml 0.1% CV (v/v) for 15 min at room temperature followed by three washes with 2.5 ml ultra-pure water and drying at 60°C. The remaining CV on the inner surface of the tube was dissolved in 1.0 ml of 33% glacial acetic acid (v/v) with vortexing, and 200 μl of the sample was transferred to a new 96-well plate and the optical density of the sample at 590 nm was measured with a microplate reader (Spectra M2; Molecular Devices, Sunnyvale, CA, United States).

### Biofilm Formation on Stainless Steel

Stainless steels (type: 304) were cut into strips (1 cm × 3 cm × 0.2 mm) and processed as described by Tapia-Rodriguez et al. (2017). An overnight culture of *H. alvei* H4 WT and *ΔluxI* were diluted 100-fold with LB medium to a cell density of about 10^6^ CFU/ml. Aliquot (10 ml) of the diluted culture was placed in a test tube (1.5 cm × 10 cm) containing a stainless-steel strip and incubated at 30°C for 24 h without shaking. After incubation, the strip was transferred to a small test tube (1.5 cm × 5 cm) containing 5 ml PBS to wash off unattached cells. This washing step was repeated three times and the strip was then transferred to a 10 ml-tube and sonicated for 1.5 min in an ultrasonication bath (power, 300 W, 37 kHz 37; Elma; Elmasonic P; Germany) to disperse the biofilm as previously described (Jahid et al., 2015). Finally, the bacterial suspension was vortexed and serially diluted with 0.85% NaCl solution, and then spread onto an LB agar plate. The plate was incubated at 30°C for 24 h and the colonies appearing on the plate were then counted.

### Biofilms Detected by SEM

*H. alvei* H4 WT and Δ*luxI* were separately incubated in LB medium for 16 h at 30°C with shaking at 150 rpm. The culture was then diluted to a final density of about 1.0 × 10^7^ CFU/ml. First, 1 ml of the diluted culture was dispensed onto a sterile stainless steel (1 cm × 1 cm × 0.2 mm) placed inside a 24-well microtiter plate and cultured at 30°C for 24 h. The biofilm deposited on the stainless steel was then was prepared for scanning electron microscopy (SEM) analysis according to the method described by Nithya et al. (2010). Briefly, the stainless steel was gently washed with sterile 0.1 M PBS, and the biofilm was fixed with 2.5% glutaraldehyde solution for 2 h followed by washing with 0.1 M PBS. The stainless steel was subjected to dehydration under a graded series of tert-butanol. This was followed by critical point drying, gold sputtering and SEM observation (Quanta 450, Waltham, MA, United States) performed at 3.0 kV under 5000 and 50000× magnifications as described previously (Hou et al., 2018).

### Swimming Motility Assay

To measure the swimming motility of *H. alvei* H4 and Δ*luxI*, motility agar (2.6 g/l agar, 10 g/l tryptone, 5 g/l NaCl) (Zhu and Winans, 1998) was used. *H. alvei* H4 or *ΔluxI* was incubated in LB medium at 30°C until the OD_600__nm_ of the cultures reached 1.0. Aliquot (3 μL) of the culture was spotted at the center of the motility agar plate followed by incubation at 30°C. The extent of motility was assessed by measuring the diameter of the zone spread from the point of inoculation. To detect the effect of AHLs on the swimming motility of *ΔluxI*, C4-HSL, C6-HSL, and 3-o-C8-HSL were added to separate motility agar plates. The final concentrations of C4-HSL, C6-HSL, and 3-o-C8-HSL in the plates were 150, 200, and 100 μg/ml, respectively. Swimming motility was determined as described above.

### Real-Time PCR Assay

*H. alvei* H4 WT and *ΔluxI* was cultured in LB medium the absence or presence of AHLs (C4-HSL, C6-HSL, and 3-o-C8-HSL) at 30°C with shaking at 150 rpm for 16 h. When the OD_600__nm_ of the cultures reached 1.65, the cells were harvested and subjected to total RNA extraction using a RNAprep Pure Bacteria Kit (DP430, TIANGEN, Beijing). About 500 ng of total RNA was reversely transcribed into cDNA using a PrimeScript^TM^ Reagent kit (RR047, Takara, Japan). The reaction mixture consisted of 2 μL template cDNA, 12.5 μL SYBR Premix Ex Taq^TM^ (RR420, Takara, Japan), 0.5 μL each of the forward and reverse primers (10 mM) (**Table 3**), and 9.5 μL RNA free water. Amplification was performed with a Step-One Thermal Cycler (Applied Biosystems, United States) and consisted of 40 cycles of denaturation at 95°C for 15 s, annealing at 95°C for 30 s, and extension at 60°C for 45 s. The *16S rRNA* gene was used as housekeeping control. The result was analyzed by 2^-ΔΔCT^ method (Schmittgen and Livak, 2008).

**Table 3:** Primers used for RT-PCR.

Primer name	Function	Primer sequences
*16S rRNA*	16S ribosomal RNA	5′-TAGCGGTGAAATGCGTAG-3′
		5′-TCGTTTACAGCGTGGACTA-3′
*flgA*	Flagella basal body P-ring formation protein	5′-GGGTTATCACCAATCCTG-3′


		5′-GGTAATGGGTTGTAAATCG-3′
*cheA*	Chemotaxis protein	5′-CCAACTTCGTCGTCGGTCATG-3′
		5′-GAACATCAGGGCGGCAAT-3′
*motA*	Flagellar motor protein	5′-TTCATGTTGCCGCTTACC-3′
		5′-ACCCGCAAGAAAGTGAAC-3′
*luxR*	AHLs receptor	5′-CTTTATTGGGCGAGTATGG-3′
		5′-TTGTCGGGCGTTGCTTAC-3′

### Statistical Analysis

Three replicate trials were carried out for each sample, and all the experiments were repeated three times. Data were analyzed by one-way analysis of variance (ANOVA) using SPSS18.0 software, and expressed as means ± standard deviations (SDs). Statistical significance was considered at the *P* < 0.05 level. All graphs were drawn with OriginPro 8.6 software.

## Results

### AHLs Production by *luxI* Mutant

**FIGURE 1:**
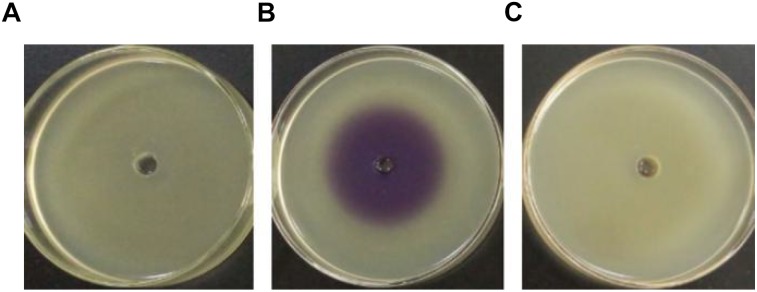
Detection of AHL production via the biosensor strain CV026. CV026 was cultured in the absence of ethyl acetate extract prepared from LB medium **(A)**, *H. alvei* H4 culture supernatant **(B)**, and Δ*luxI* culture supernatant **(C)**.

Pigment production assay showed a violet zone in the center of the plate when CV026 was incubated with the ethyl acetate extract prepared from the culture supernatant of *H. alvei* H4 but not from the culture supernatant of *ΔluxI* (**Figure 1**), indicating the lack of AHLs production by *ΔluxI*. The assay demonstrated the dependence of AHLs production on a functional *luxI* gene.

### Response of *luxR* to AHLs

**FIGURE 2:**
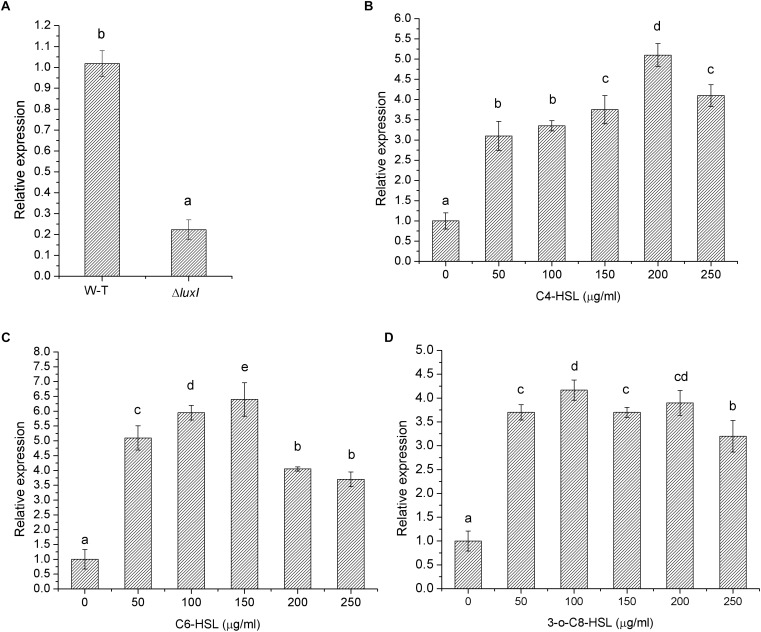
Relative expression of the *luxR* gene by *H. alvei* H4 cultured in the absence and presence of AHLs. **(A)** *H. alvei* H4 and Δ*luxI* cultured in the absence of AHLs. Δ*luxI* cultured in the presence of C4-HSL **(B)**, C6-HSL **(C)**, and 3-o-C8-HSL **(D)**. The mRNA level of the *luxR* gene was determined by *q*RT-PCR. Data are the means ± SEMs (*n* = 3). Different letters above the columns indicate significant differences at the *P* < 0.05 level.

The effect of *luxI* mutation was further investigated by measuring the level of *luxR* mRNA in *ΔluxI* and compared it with that of WT strain. Δ*luxI* exhibited about fivefold reduction in the level of *luxR* mRNA relative the WT (**Figure 2A**), which clearly suggested that the expression of the *luxR* gene in the mutant was significantly (*P* < 0.05) inhibited. However, in the presence of exogenous AHLs, the level of *luxR* mRNA gradually increased with increasing concentrations of AHLs, suggesting that the expression of *luxR* could be stimulated by the presence of AHLs (**Figure 2B**). Maximum increase in *luxR* mRNA level stimulated by AHL ranged from 5.5-fold in the case of C4-HSL to 6.5-fold in the case of C6-HSL, while 3-o-C8-HSL yielded somewhat lower increase (4.5-fold). Thus, C6-HSL appeared to exert the strongest stimulatory effect on the expression of *luxR*.

### Biofilm Formation

**FIGURE 3:**
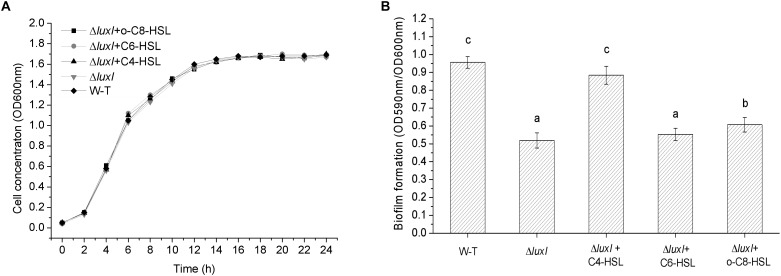
Growth curve and biofilm formation of wild type (WT) *H. alvei* H4. **(A)** Growth curve; **(B)** Biofilm formation. Wild type *H. alvei* H4 was cultured in the absence of AHL only. *ΔluxI* was cultured both in the absence and presence of the indicated AHLs (150 μg/ml C4-HSL, 200 μg/ml C6-HSL and 100 μg/ml 3-o-C8-HSL), respectively. Data are the means ± SEMs (*n* = 3). Different letters above the columns indicate differences at the *P* < 0.05 level.

Growth of *H. alvei* H4 was not affected by the deletion of the *lux*I gene, as shown by the similar growth curves between WT and *ΔluxI* in the absence of AHLs (**Figure 3A**). Furthermore, the addition of AHLs to the culture of *ΔluxI* also had not obvious effect on its growth. This suggested that growth of the bacterial cells was not dependent on the product of the *luxI* gene. However, deletion of the *luxI* gene had a significant effect on biofilm formation by *H. alvei* H4, as shown by the significantly higher level of biofilm formed by the wild type (WT) compared with Δ*luxI* (**Figure 3B**). Addition of AHLs to the culture of Δ*luxI* appeared to cause increase in biofilm formation. The extent of biofilm formation of Δ*luxI* in the presence of C4-HSL was significantly (*P* < 0.05) enhanced and almost similar to that of the WT strain. However, C6-HSL seemed to have no effect on the biofilm formation of Δ*luxI*, causing no significant increase. Obvious promotion (*P* < 0.05) of biofilm formation on Δ*luxI* were also achieved at the presence of 3-o-C8-HSL, but still far less than that of C4-HSL on Δ*luxI* and WT strain. The result suggested that C4-HSL could be the AHL with major influence on biofilm formation.

### Biofilms on Glass

**FIGURE 4:**
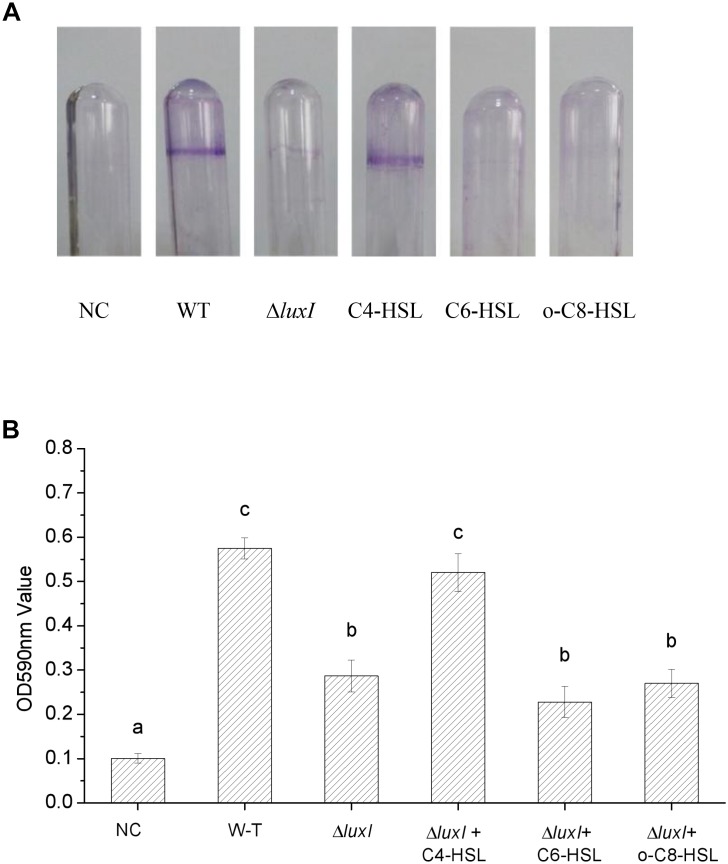
Biofilm formation by *H. alvei* strains. The image shows the biofilms formed on glass surface by WT and Δ*luxI* with and without AHLs. The plot shows the extent of biofilm formed on the glass surface as quantitated by OD_590nm_. NC, negative control. Data are the means ± SEMs (*n* = 3). Different letters above the columns indicate differences at the *P* < 0.05 level.

An intense zone of purple stain was found on the wall of the glass tube used to culture WT strain, indicating the presence of biofilm (**Figure 4A**). On the other hand, only very slight purple staining was found on the wall of the glass tube that was used to culture Δ*luxI*, suggesting a lack of biofilm being formed by Δ*luxI*. Addition of C4-HSL to the Δ*luxI* culture resulted in obvious enhancement of the purple zone, but this did not occur when either C6-HSL or 3-o-C8-HSL was added to the culture. The result clearly suggested that the capacity of Δ*luxI* to form biofilm was greatly reduced compared with its WT strain counterpart, and that only C4-HSL was able to restore its biofilm formation capacity.

### Biofilms on Stainless Steel

**FIGURE 5:**
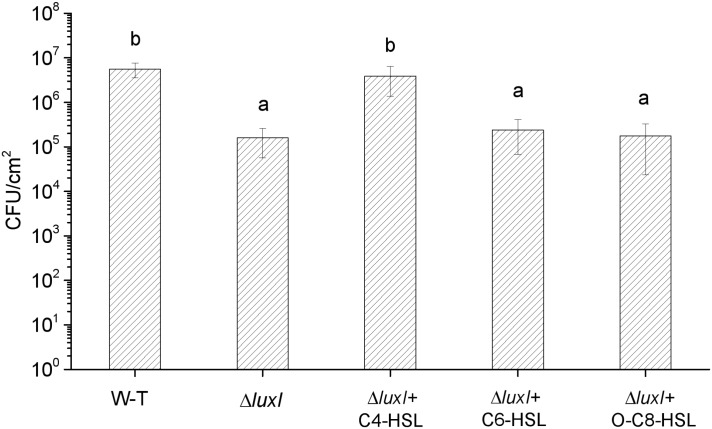
Biofilm dormation of *H. alvei* H4 on stainless steel surface. *H. alvei* H4 and its mutant Δ*luxI* were allowed to form biofilm on stainless-steel surface with and without AHLs (C4-HSL, C6-HSL, and o-C8-HSL). The cells were then washed and counted. Data are the means ± SEMs (*n* = 3). Different letters above the columns indicate significant differences at the *P* < 0.05 level.

The mutant Δ*luxI* was also compared with its WT counterpart in term of the ability to form biofilm on the surface of stainless steel. Significantly (*P* < 0.05) less colonies of Δ*luxI* were found on the surface of the stainless steel compared with the WT strain, but when Δ*luxI* was cultured in the presence of C4-HSL, the number of colonies found on the surface of the stainless steel was significantly (*P* < 0.05) increased, and being comparable with that of WT strain (**Figure 5**). However, as in the case of biofilm formed on glass surface, C6-HSL and 3-o-C8-HSL had no obvious effect on the ability of Δ*luxI* to form biofilm on stainless steel surface.

### Biofilm by SEM

**FIGURE 6:**
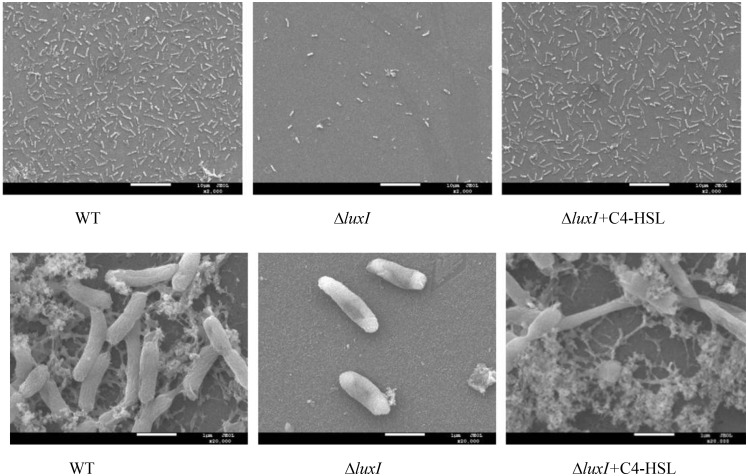
Scanning electron micrographs showing the appearance of biofilms formed by wild type *H. alvei* H4, Δ*luxI*, and Δ*luxI+*C4-HSL.

Biofilm formation of *H. alvei* H4 WT and Δ*luxI* on stainless-steel surface was detected by SEM. Obvious reduction in the number of Δ*luxI* cells on stainless-steel surface was observed, but the number of Δ*luxI* cells was restored when supplied with 150 μg/ml C4-HSL (**Figure 6**). In addition, WT cells appeared to aggregate and adhered to the surface, and produced evident EPS filaments. In contrast, *ΔluxI* tended to adhere to the surface as individual cells. When Δ*luxI* was exposed to 150 μg/ml C4-HSL, the cells also formed aggregates and adhered to the surface of stainless steel, these cells also produced a lot of EPS.

### Swimming Motility

**FIGURE 7:**
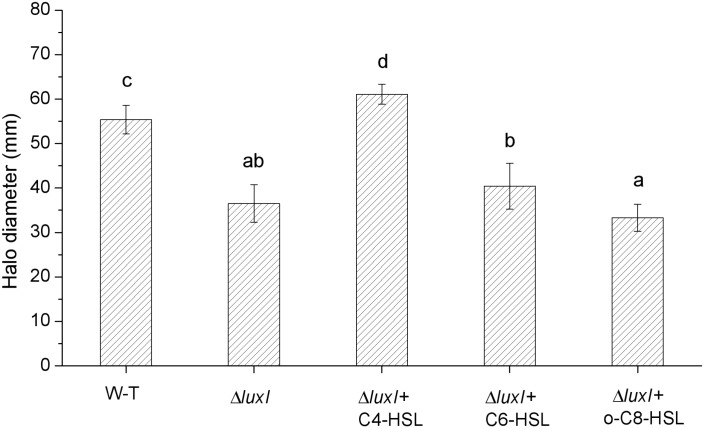
Swimming motility of wild type and mutant *H. alvei* H4. Swimming motility was conducted in the absence or exogenous AHLs in the case of WT or both in the absence and presence of AHLs in the case of Δ*luxI*. The concentrations of C4-HSL, C6-HSL, o-C8-HSL used were 150, 200, and 100 μg/ml respectively. Data are the means ± SEMs (*n* = 3). Different letters above the columns indicate significant differences at the *P* < 0.05 level.

As shown in **Figure 2B–D**, the concentrations of AHLs, which induce the relative expression of *luxR* gene to the maximum respectively, were chosen for swimming motility assay, and swimming motility was determined by the expansion of the bacterial colony from the point of application to a greater diameter on the surface of an agar plate. Compared with WT, Δ*luxI* suffered significant (*P* < 0.05) loss in swimming motility in the absence of exogenous AHLs (**Figure 7**). However, upon addition of C4-HSL, the swimming motility of *ΔluxI* increased and became significantly higher than the level of WT. Addition of C6-HSL also had some enhancing effect on the swimming motility of Δ*luxI*, but it remained obviously reduced compared with WT. In contrast, the addition of 3-o-C8-HSL appeared to have no real effect on the swimming motility of Δ*luxI*.

### Expression of *flgA*, *motA*, and *cheA*

**FIGURE 8:**
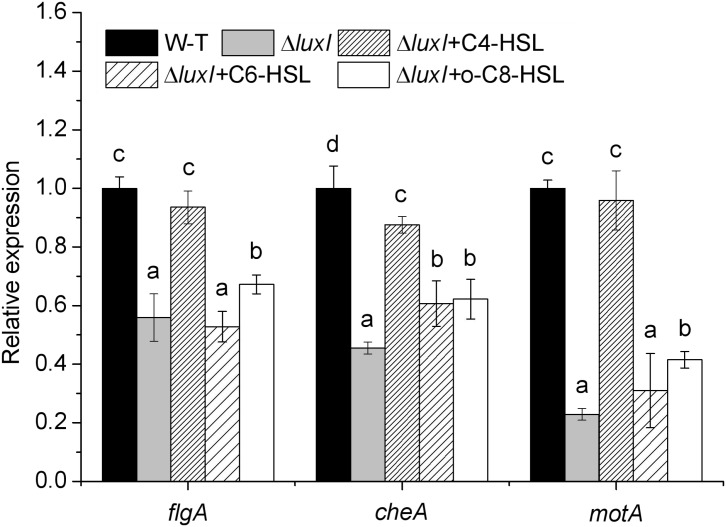
Relative expression of several genes related to biofilm formation and swimming motility of *H. alvei* H4. Expression of the *flgA*, *motA*, and *cheA* genes in WT cultured in the absence of exogenous AHL was compared with those of Δ*luxI* mutant cultured in the absence and in the presence of 150 μg/ml C4-HSL, 200 μg/ml C6-HSL, or 100 μg/ml 3-o-C8-HSL. The mRNA levels of the genes were measured by *q*RT-PCR. Data are the means ± SEMs (*n* = 3). Different letters above the columns indicate significant differences at the *P* < 0.05 level.

To further study the molecular mechanism of quorum sensing on biofilm formation, three biofilm formation and swimming motility related genes, *flgA*, *motA* and *cheA*, were chosen for analysis using *q*RT-PCR. The mRNA levels of all three genes in Δ*luxI* in the absence of exogenous AHLs were significantly (*P* < 0.05) reduced compared with those of the WT, with *motA* being the most severely affected gene (**Figure 8**). Addition of C4-HSL (150 μg/ml) to the Δ*luxI* culture restored the mRNA levels of the three genes to the levels comparable with those of the WT strain, except for the *cheA* gene, which showed lesser, but still significant increase. Similarly, the addition of C6-HSL (200 μg/ml) had some enhancing effect on the mRNA levels of *motA* and *cheA*, whereas the addition of 3-o-C8-HSL (100 μg/ml) appeared to increase the mRNA levels of all three genes, but the increases still fell short of those achieved by C4-HSL. The result suggested that AHL could stimulate the expression of the *motA* and *cheA* genes, and to a lesser extent, the expression of the *flgA* gene.

## Discussion

Mutant strain of *H. alvei* H4 with defective *luxI* gene was successfully constructed in this study, and the effect of this mutation on the quorum-sensing system was characterized with respect to AHL production, biofilm formation, and swimming motility. The *luxI* gene was generally recognized as AHL synthase gene, which controls the synthesis of AHLs (Waters and Bassler, 2005; Morohoshi et al., 2007). Indeed, the *luxI* mutant produced no detectable AHLs as assayed with the biosensor strain CV026 (**Figure 1**), consistent with the results reported for *Aeromonas hydrophila* and *Acinetobacter nosocomialis* (Khajanchi et al., 2009; Oh and Choi, 2015). The complementing strain of *luxI* was also constructed, and no differences in AHLs production, biofilm formation and swimming motility were found between comp-*ΔluxI* and WT strain (Supplementary Figure 1).

It is known to us that when local concentration of AHLs is high enough, AHLs would diffuse back into the cell, and induce the expression of *luxR* gene (Waters and Bassler, 2005; Bai and Rai, 2011). Expression of the *luxR* gene in the AHL-deficient strain of *H. alvei* H4 was found to decrease significantly, suggesting that expression of the *luxR* gene might require induction by AHLs. This hypothesis was confirmed by significant increase in *luxR* expression in the mutant when it was cultured in the presence of exogenous AHL (C4-HSL, C6-HSL, or 3-o-C8-HSL). The stimulating effect on *luxR* gene increased with the increase of AHLs concentration, causing the increase in *luxR* gene expression consequently. Similar result has also been obtained for *Aliivibrio fischeri*, whereby expression of the *luxR* gene was observed in the presence of 3-o-C6-HSL, with higher concentrations of the compound leading to more expression of the gene (Ramalho et al., 2016). In addition, there is also a threshold concentration of AHLs, and QS regulon is depressed when the concentration of AHLs exceeds this threshold, as a result of causing the reduction of *luxR* gene expression (Swift et al., 2001). Similar changes of *sinR* gene expression affected by AHLs were also obtained by Gao et al. (2012) in *Sinorhizobium meliloti*. Besides, response of CviR to autoinducer were found depressed when exposed to high concentration AHLs (Swem et al., 2009) in *C. violaceum*, consistent to the results achieved in the present study (**Figure 2B–D**).

Biofilm formation by the *luxI* mutant was inhibited, and since the mutant was deficient in AHL, this indicated that biofilm formation might be modulated by AHLs, and is associated with AHL-mediated QS. As reported by Ammor et al. (2008), bacterial phenotype such as resistance to antimicrobial compounds, biofilm formation, bioluminescence; pigment production, virulence gene expression, swimming motility and production of degradative extracellular proteases are regulated by the QS system. Furthermore, the addition of C4-HSL to the *ΔluxI* culture restored the ability of the mutant cells to form biofilm, while the addition of C6-HSL and 3-o-C8-HSL appeared to have no significant effect, suggesting that the C4-HSL might play an important role in the regulation of biofilm formation in *H. alvei* H4. Similar result has been reported for *A. hydrophila*, in which biofilm formation and protease activity, which are inhibited in an AHL-deficient strain, can be stimulated by the addition of C4-HSL to the culture (Khajanchi et al., 2009). Niu et al. (2008) investigated the regulatory role of C12-HSL in the biofilm formation of *Acinetobacter baumannii* M2 and showed that biofilm formation is obviously inhibited in an *abaI* mutant, but can be restored by the addition of C12-HSL to the culture. Similarly, in *Acinetobacter* sp. strain DR1, C12-HSL was also found to play the same role as in *A. baumannii* M2 (Kang and Park, 2010). In *P. aeruginosa*, the LasR protein (a LuxR homologous protein) is soluble and stable only when it is produced in the presence of its cognate AHL ligand 3-o-C12-HSL, as the protein is becoming less soluble if it is produced in the presence of a different AHL, such as 3-o-C8-HSL or 3-o-C6-HSL, while soluble form is not produced in the presence of C4-HSL (Bottomley et al., 2007). Hence, C4-HSL might be the cognate ligand of LuxR in *H. alvei* H4, and it may possibly bind to the LuxR protein and play a regulatory role. In *P. syringae* and *S. meliloti*, the production of extracellular polysaccharide has been shown to be regulated by QS (Marketon et al., 2003; Quiñones et al., 2005). Therefore, the reduction in the numbers Δ*luxI* cells adhering to polystyrene, glass and stainless-steel surfaces that we observed (Figure 3–5) might be caused by the reduction in biofilm formation and extracellular polysaccharide secretion. According to the results achieved in the present research and the studies reported by Nasser et al. (1998) and Andersson et al. (2000), different AHLs in bacteria may regulate different phenotypes, for *H. alvei* H4, C4-HSL seems to have a more important effect on regulating biofilm formation and swimming motility than C6-HSL and 3-o-C8-HSL, however, in other phenotypes regulated by quorum sensing system, C6-HSL and 3-o-C8-HSL might act as regulators.

Another important factor in the biofilm formation of bacteria is the extent of swimming motility, which contributes to the early development of the biofilm architecture (microcolony formation) (Srey et al., 2013). Bacterial motility depends mainly on the movement of flagella and Brownian motion (Li et al., 2008). Furthermore, some genes such as the *flgA*, *motA*, and *cheA* genes have been shown to be involved in biofilm formation and swimming motility (Lee et al., 2015). The data on swimming motility assay and *q*RT-PCR analysis of the expression of these three motility-related and biofilm-related genes, *flgA*, *motA*, and *cheA* (**Figure 8**), appeared to suggest that swimming motility might be regulated by regulating the expression of these genes. Since Δ*luxI* exhibited significantly less swimming motility and lower expression of the *flgA*, *motA*, and *cheA* genes than WT strain in the absence of exogenous AHLs, these two factors might also be the cause of poor biofilm formation displayed by the mutant. Similar results have also been reported by Gurich and González (2009), whereby deletion of the *sinI* gene in *S. meliloti* can result in significant reduction in the expression of the motility genes *flaA* and *flaB*, but such reduction can be reversed by the presence of AHLs. In contrast to our data, Wang T. et al. (2016) demonstrated that in *Acidovorax citrulli*, biofilm formation, motility and adherence of bacterial cells to a solid surface can be significantly promoted when the *accR/I* gene is defective. Furthermore, in *A. nosocomialis*, both biofilm formation and motility were found to be modulated by the *luxR* homologous gene *anoR* (Oh and Choi, 2015), and not the *anoI* gene. Therefore, the regulation of QS in biofilm formation their swimming motility might be different for different bacteria.

## Conclusion

In *H. alvei*, AHL-mediated QS system plays a key part in swimming motility and biofilm formation on different solid surfaces. We have shown here that among the different AHLs tested, C4-HSL appeared to exert a more significant impact on the modulation of these properties of the cells. Further study focusing on the regulation mechanism of the *luxR/I* gene, and the screening of effective QS inhibitors for *H. alvei*, and the application of these inhibitors to food production may bring about economic benefits as well as preventing the spread of food-related incident of infection among the public.

## Author Contributions

HMH and GZ designed this study. YZ conducted the experiments. YZ, HMH, and YW performed the data analyses. HSH, GZ, and YZ drafted and revised the manuscript. All authors read and approved the final version of this manuscript.

## Conflict of Interest Statement

The authors declare that the research was conducted in the absence of any commercial or financial relationships that could be construed as a potential conflict of interest.
