# Supplementary material for: AHLs Regulate Biofilm Formation and Swimming Motility of Hafnia alvei H4
Source: Front Microbiol. 2019 Jun 19;10:1330. doi: 10.3389/fmicb.2019.01330 (PMC6593095; doi:10.3389/fmicb.2019.01330)
Supplement: Supplementary file 1 [file Table_1.DOCX]

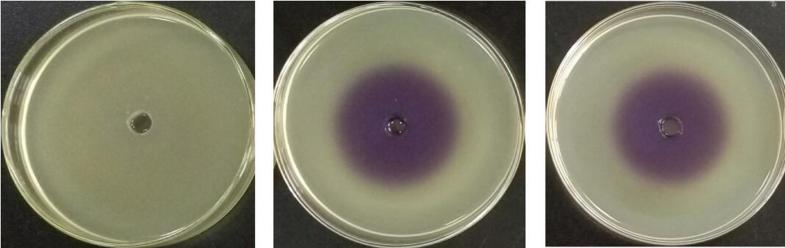


A

(a) (b) (c)

B

C

E

D

FIGURE 1. Detection of (A) AHL production via the biosensor strain CV026. CV026 was cultured in the absence of ethyl acetate extract prepared from (a) LB medium, (b) *H. alvei* H4 culture supernatant, and (c) comp-∆*luxI* culture supernatant. (B) Growth curve, (C) biofilms on 96-well plate, (D) biofilms on stainless-steel and (E) swimming motility of wild-type *H. alvei* H4 and comp-∆*luxI* strain. Data are the means ± SEMs (n=3). Different letters above the columns indicate differences at the *P*< 0.05 level.
